# Mitogenomes from The 1000 Genome Project Reveal New Near Eastern Features in Present-Day Tuscans

**DOI:** 10.1371/journal.pone.0119242

**Published:** 2015-03-18

**Authors:** Alberto Gómez-Carballa, Jacobo Pardo-Seco, Jorge Amigo, Federico Martinón-Torres, Antonio Salas

**Affiliations:** 1 Unidade de Xenética, Departamento de Anatomía Patolóxica e Ciencias Forenses, and Instituto de Ciencias Forenses, Facultade de Medicina, Universidade de Santiago de Compostela, 15872, Galicia, Spain; 2 Grupo de Investigación en Genética, Vacunas, Infecciones y Pediatría (GENVIP), Hospital Clínico Universitario and Universidade de Santiago de Compostela (USC), Galicia, Spain; 3 Pediatric Emergency and Critical Care Division, Department of Pediatrics, Hospital Clínico Universitario de Santiago, Santiago de Compostela, Galicia, Spain; University of Perugia, ITALY

## Abstract

**Background:**

Genetic analyses have recently been carried out on present-day Tuscans (Central Italy) in order to investigate their presumable recent Near East ancestry in connection with the long-standing debate on the origins of the Etruscan civilization. We retrieved mitogenomes and genome-wide SNP data from 110 Tuscans analyzed within the context of The 1000 Genome Project. For phylogeographic and evolutionary analysis we made use of a large worldwide database of entire mitogenomes (>26,000) and partial control region sequences (>180,000).

**Results:**

Different analyses reveal the presence of typical Near East haplotypes in Tuscans representing isolated members of various mtDNA phylogenetic branches. As a whole, the Near East component in Tuscan mitogenomes can be estimated at about 8%; a proportion that is comparable to previous estimates but significantly lower than admixture estimates obtained from autosomal SNP data (21%). Phylogeographic and evolutionary inter-population comparisons indicate that the main signal of Near Eastern Tuscan mitogenomes comes from Iran.

**Conclusions:**

Mitogenomes of recent Near East origin in present-day Tuscans do not show local or regional variation. This points to a demographic scenario that is compatible with a recent arrival of Near Easterners to this region in Italy with no founder events or bottlenecks.

## Background

Etruria comprised the area located between the Arno and the Tiber Rivers, corresponding roughly to present-day Tuscany, western Umbria, and northern Latium in Italy. The Etruscan civilization rose in this region around 800 BC, marking a transition from the preceding Iron Age Villanovan culture. The Etruscans developed advanced transport infrastructure, implemented agriculture in the region and produced, among other feats, well-known figurative art and sophisticated metalwork. The origin of the Etruscans has largely been lost in prehistory. Since no original Etruscan texts have been recovered (e.g. literature, religion or philosophy), much of what is known about them is derived from archaeological findings such as grave goods. Two main theories on their origin have been intensively debated among experts. On the one hand, the ancient Greek historian Herodotus (484–425 BC) speculated that the Etruscans were migrants from the Western coast of Anatolia (a region called Lydia). On the other hand, numerous modern historians have been very skeptical of this theory, and many of them have proposed that the Etruscan civilization developed *in situ* from an indigenous population.

In order to shed further light on the mysterious origin of the Etruscans, the present-day inhabitants of Tuscany have been the focus of a number of genetic studies, under the assumption that they could have retained some of the genetic legacy of their presumable ancient Etruscan ancestors. Most of the genetic studies carried out to date on the origin of the Etruscans have been conducted on the analysis of the mitochondrial DNA (mtDNA) control region. Thus, in the late nineties, Francalacci et al. [1] analyzed the mtDNA control region of 49 Tuscans, and showed for the fist time the intermediate position of Tuscan mtDNAs between sequences from Europe and the Near East. Since then, a number of genetic studies of modern and ancient DNA [2–8] have corroborated the presence of a significant Near East component in modern Tuscans. For instance, Achilli et al. [8] and Brisighelli et al. [3] reported the presence of Near East haplotypes in 5 to 10% of modern Tuscans, with a peak of 18% in the ‘Etruscan’ village of Murlo [8]. Following the same line of evidence, one study based on *Bos Taurus* mtDNAs [9] showed that Tuscan bovines were genetically closer to Near Eastern than to European gene pools, bearing out the hypothesis of the arrival to Central Italy of Eastern settlers together with their cattle in the Late Bronze Age.

A few genetic studies have focused on the analysis of human remains. Vernesi et al. [2] obtained the mtDNA hypervariable region I (HVS-I) of skeletons from ten Etruscan necropoleis dated to between the 7^th^ and the 3^rd^ centuries BC; these authors reported for the Etruscans a closer evolutionary relationship with the eastern Mediterranean shores than for modern Italian populations. Based on the analysis of 27 medieval Tuscans, Guimaraes et al. [4] claimed the existence of extensive demographic changes occurring before AD 1000 in Tuscany that would explain the differences between contemporary and medieval mtDNAs from Tuscany. Analysis of previously generated HVS-I data from modern and ancient DNA combined with demographic simulations led Tassi et al. [7] to the conclusion that the links between Tuscany and Anatolia dated back to a remote stage of prehistory which could be traced to the spread of farmers during the Neolithic (> 6,500 years ago [y.a.]). Finally, Ghirotto et al. [5] analyzed the HVS-I segment extracted from 14 individuals buried in two Etruscan necropoleis, and concluded that the genetic links between Tuscans and Anatolia dated back to at least 5,000 y.a.

Therefore, while Brisighelli et al. [3] dated the arrival of the Near East U7a2a haplogroup (now known to as U7b1; Phylotree Build 16 [10]) to the Isle of Elba about 2,300 y.a., and the studies of Achilli et al. [8] and Pellecchia et al. [9] agreed with a recent arrival of Near Easterners to Tuscany (thus supporting the Herodotus theory), other studies (mainly based on the analysis of ancient DNA combined with demographic simulations) favored the proposition of a connection between Tuscans and the Near East in the Neolithic (that is, the moment in which gene flow was extensively occurring in Europe). The latter option would suggest that the Etruscan culture developed locally in Italy, and not as a consequence of the arrival of immigrants from Eastern Mediterranean regions.

At the same time, while some mtDNA studies suggest Anatolia (present-day Turkey) [4,11] or the southern Mediterranean [2] as the more likely regions of origin for Tuscan Near East haplogroups, the genome-wide study carried out by Pardo-Seco et al. [11] suggests a primary origin for this Near East component in the South Caucasus. Their demographic model aimed to accommodate the different genetic findings by proposing a multi-step demographic process: this includes an ancient origin for the proto-Etruscans in the region of present Iran, followed by a population expansion in the South Caucasus, and from here westwards to the West Mediterranean shores of Turkey. Finally, this Near East population could have crossed the Mediterranean basin towards Central Italy and eventually given raise to the Etruscan civilization about 3,000 y.a. Such a model would also be coherent with Herodotus’s theory that considered West Turkey as the most likely origin for the Etruscans.

By analyzing new mtDNA data from Tuscany, it may be possible to find evidence to verify the presence of Near East components in this region. The present study goes beyond previous attempts, as this is the first time that entire mtDNA genomes at a population level are analyzed in this long-debated historical context.

## Material and Methods

### Data mining from The 1000 Genome Project data

For the bioinformatics treatment of The 1000 Genome Project (http://ftp.1000genomes.ebi.ac.uk/vol1/ftp/phase1/data) we took advantage of previous bioinformatic developments [12,13]. All data files were first downloaded from the project’s public site; a script was used to retrieve the mitochondrial genome only from the bam alignments available through “samtools view” calls. The resulting reduced bam files were then indexed through “samtools index”, and then processed using the protocol suggested in the GATK best practice for variant detection site (http://www.broadinstitute.org/gatk/guide/best-practices): the duplicate reads were first removed, the remaining reads were realigned around known and candidate indel sites and their quality scores per base were recalibrated, and the HaplotypeCaller algorithm was ultimately used to detect all the variants. GATK v2.5 was used, along with all its bundle files for version b37 of the reference genome. The variants obtained using the GATK pipeline on The 1000 Genome Project data are in VCF format, which is reformatted using an in-house script looking for transitions, transversions, insertions and deletions, in order to output them all in the standard mtDNA nomenclature that allows each sample’s variability information (mtDNA haplotypes) to be summarized into a single text line.

S1 Table shows all the annotated mitogenomes from Tuscans and a comparison of these annotated variants with the annotations carried out by Zheng et al. [14] using the same dataset. S2 Table contains information on coverage and other parameters generated by the annotated software for the variants of the mitogenomes extracted from The 1000 Genome Project.

Annotation of variants was performed using the rCRS as the reference sequence [15,16]. From The 1000 Genome Project raw data we obtained coverage and mapping quality information per mtDNA position/variant. The average coverage with mapping quality above zero of the Tuscan data was very high (mean DP value = 2408.18; SD = 850.167).

### Statistical analysis

The phylogenetic reconstruction of mitogenomes was carried out by building maximum parsimony trees and fitting the trees to the generally accepted worldwide phylogeny in PhyloTree Build 16 (http://www.phylotree.org; [10]). Trees were drawn with the assistance of Haplogrep 2.0 Beta version (http://haplogrep.uibk.ac.at/blog/visualize-yo/) and supervised manually. The time to the most recent common ancestor (TMRCA) for the U7a4a1a clade was calculated using the maximum likelihood (ML) procedure with PAML 3.13 [17] and a set of outgroups belonging to other U7 clades and L3. We additionally computed the averaged distance (ρ) of all haplotypes in a clade to the respective root haplotype [18], and heuristic estimates of the standard error (σ) were calculated from an estimate of the genealogy [19]. Hotspot mutations such as the transitions T16182C, T16183C and T16519C [20] were excluded from the calculations. Mutational distances were converted into years using the corrected evolutionary rate proposed by Soares et al. [20].

We used Haplogrep (http://haplogrep.uibk.ac.at) for exploratory haplogrouping, but the final haplogroup classification was manually checked according to recommendations [21]. A large database of >26,000 entire genomes representing worldwide populations was used for comparison. Control region profiles were searched in different public databases (EMPOP: http://empop.org; mitoSearch: http://www.mitosearch.org, Sorenson: http://www.smgf.org/pages/mtdatabase.jspx); and an in-house database of public literature (>180,000 control region profiles).

The Arlequin v3.5 [22] software was used for the computation of different molecular diversity indices, including haplotype (*HD*) and nucleotide (*π*) diversities, the mean number of pairwise differences (*M*) (S3 Table) and *F*_*ST*_ (S4 Table). *F*_*ST*_ were computed using the few mitogenome population datasets available from Europe, South Caucasus and the Near East, and these distances were used to carry out a Classical Multidimensional Scaling (MDS) in order to discriminate clusters of genetic variation. Diversity indices, when estimated from control region segments, were computed using the sequence range 16024 to 16365, since this is the common segment reported in the literature for the different datasets. Moreover, for these calculations, the problematic variation around position 16189, which is usually associated with length heteroplasmy (e.g. 16182C or 16183C), was ignored.

The spatial geographical representation of haplogroup frequencies and nucleotide diversities (*π*) was carried out using SAGA v. 2.1.1 (http://www.saga-gis.org/). We followed the commonly used ordinary Kriging method for interpolating frequency values; other interpolated methods yielded virtually the same results.

In-house R (http://www.r-project.org) and Perl (http://www.perl.org) scripts were used to display results obtained from the different software packages used.

## Results

### Tuscan mitogenomes

By adding 110 Tuscan mitogenomes (S1 Table) to the worldwide phylogeny, we identified 29 hitherto un-described new haplogroups and sub-haplogroups represented by at least two different genomes and differing by at least one mutational stable position [20].

By investigating a large database of worldwide mitogenomes and control region data, we were able to identify those haplotypes in Tuscans that probably had a Near East origin in a recent period.

**Fig 1:**
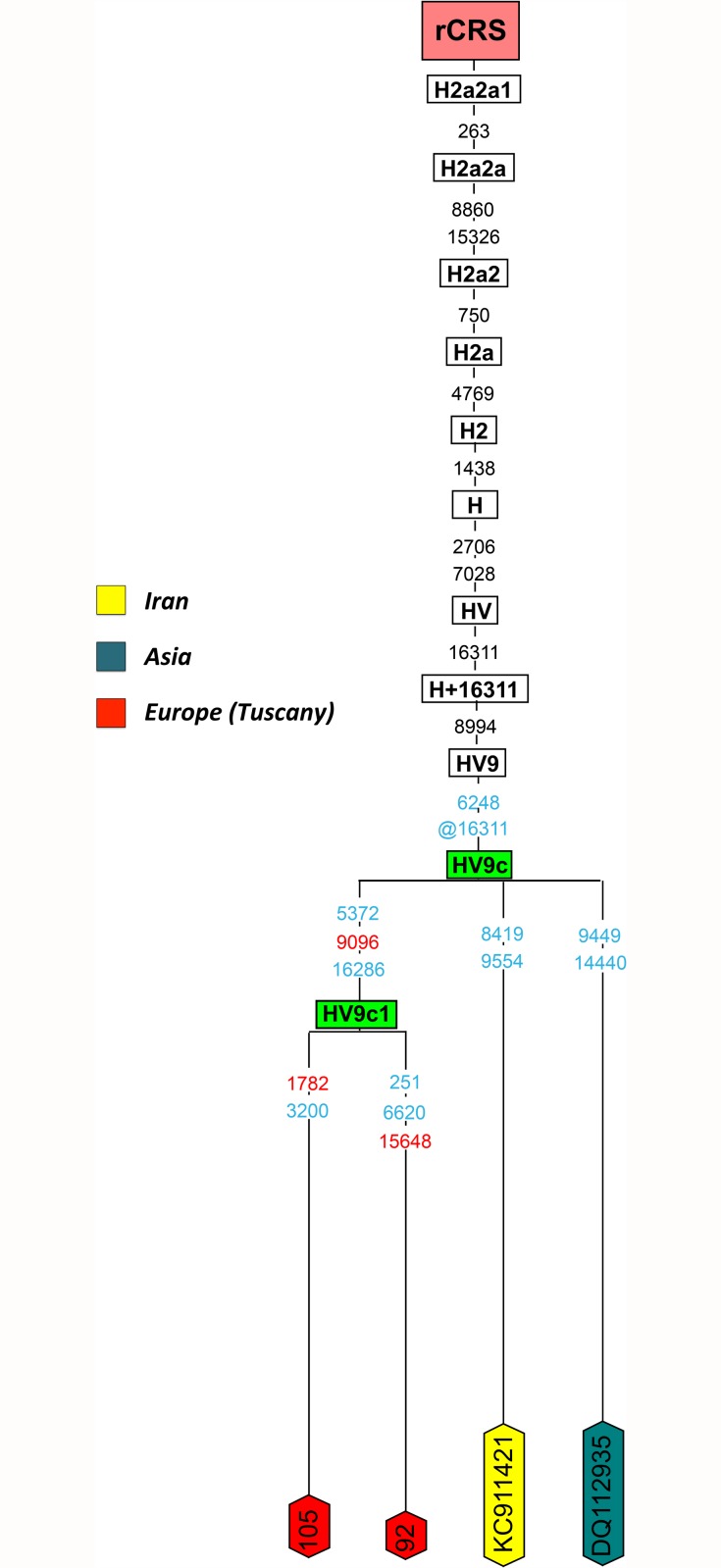
Maximum parsimony tree of haplogroup HV9c. All the specifications below are common for all the phylogenetic trees built for the present study, although not all of them necessarily concur in the same tree. The position of the revised Cambridge Reference Sequence (rCRS) is indicated for reading off sequence motifs [15]. Mitochondrial DNA variants are indicated along the branches of the phylogenetic tree. Mutations are transitions unless a suffix A, C, G, or T indicates a transversion. Other possible suffixes indicate insertions (.) and deletions (d). The prefix ‘@’ indicates a back or missing mutation. Mutational hotspot variants at positions 16182, 16183, and 16519, indels at 515–524, additions at 16193 as well as variation around position 310 and length or point heteroplasmies were not considered for the phylogenetic reconstruction. Polymorphisms colored in blue at the tips of the phylogeny are private variants while polymorphisms colored in red are private variants not described in PhyloTree Build 16. Haplogroup green boxes indicate a new haplogroup or a difference with respect to haplogroup definitions in PhyloTree Build 16.

The Tuscan haplotypes #92 and #105 belong to haplogroup HV9. These sequences, together with two other mitogenomes (KC911421 from Iran and DQ112935 from a Bedouin sampled in Asia) determine the sub-clade HV9c (Fig. 1), here defined only by T6248C and a reversion at position 16311. The two Tuscan samples define a nested subclade, namely HV9c1. When the complete genome database is inspected, it is observed that HV9 is a rare haplogroup that is mainly present in North and East Europe (*n* = 27; S3 Table); however, the sub-clade HV9c has been observed only in the Near East and in Tuscany.

**Fig 2:**
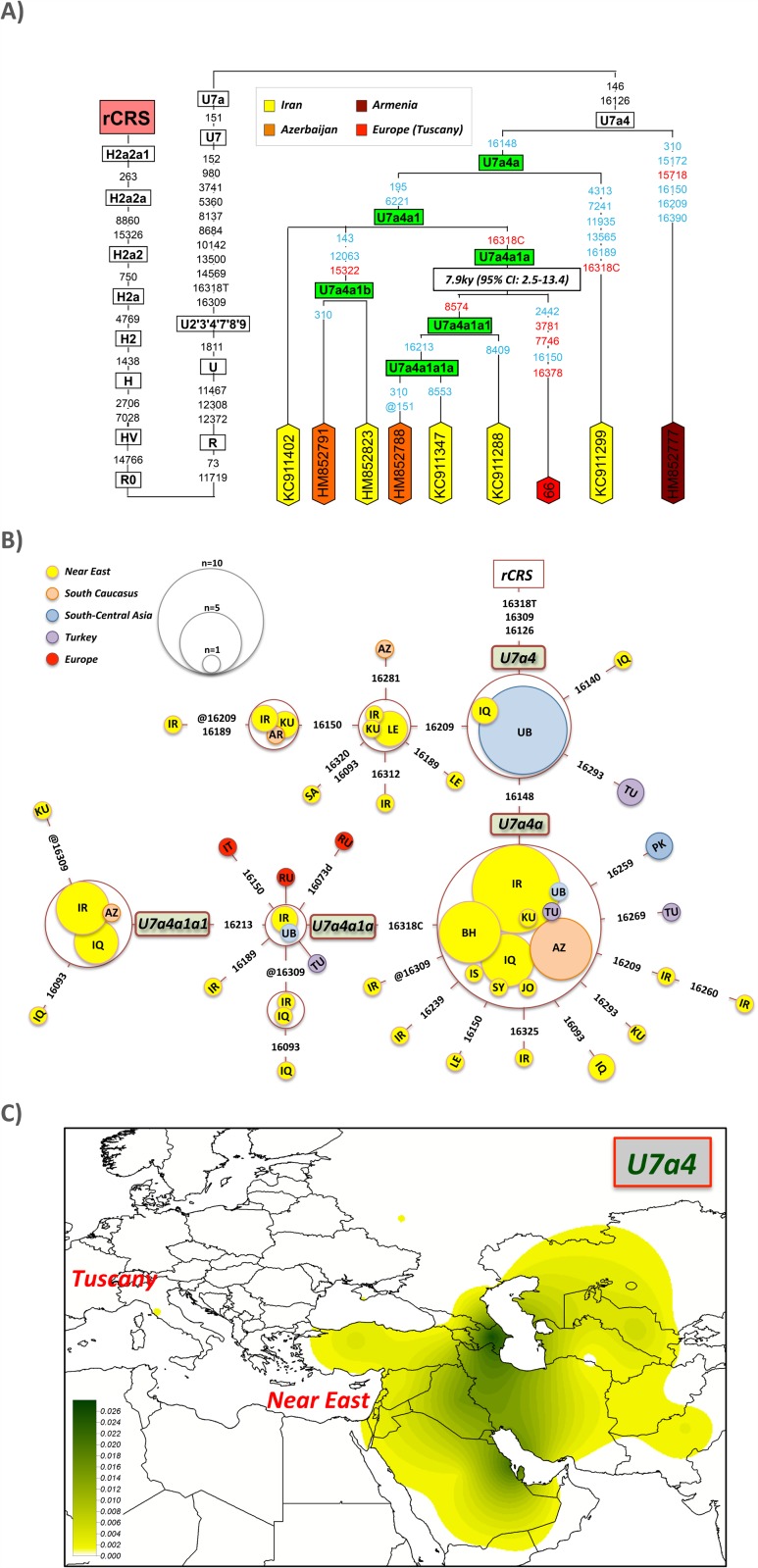
Maximum parsimony tree of haplogroup U7a4 mitogenomes and TMRCA of the clade U7a4a1a using PAML (A) and network of U7a4 HVS-I (positions between 16024–16365) sequences (B). Population codes are as follows: IR = Iran, IQ = Iraq, KU = Kuwait, AR = Armenia, LE = Lebanon, SA = Saudi Arabia, TJ = Tajikistan, TN = Tunisia, IT = Italy, TU = Turkey, PK = Pakistan, RU = Russia, AZ = Azerbaijan, BH = Bahrain, JO = Jordan, SY = Syria, IS = Israel, and UB = Uzbekistan. The map in (C) shows the geographic distribution of haplogroup U7a4 interpolated frequencies; see also S1 Fig.

Haplotype #66 is particularly interesting (Fig. 2). It belongs to the typical Near Eastern haplogroup U7, the same haplogroup found previously in the Isle of Elba at high prevalence [3], but representing a different sub-branch. U7 has two main haplogroups, U7a and U7b. The Tuscan U7 haplotype belongs to U7a, in particular, to the branch U7a4 determined by transitions T146C and T16126C. Eight out of the nine U7a4 mitogenomes were sampled in the Near East (five in Iran) or South Caucasus (one in Armenia and two in Azerbaijan); the ninth haplotype is the Tuscan #66 (Fig. 2A). U7a4 can be further resolved into a series of new nested branches defined by the following variants: U7a4>U7a4a (C16148T), U7a4a>U7a4a1 (T195C-T6221C), U7a4a1>U7a4a1a (A16318C), U7a4a1a>U7a4a1a1 (C8574T), U7a4a1a1>U7a4a1a1a (G16213A) and, U7a4a1>U7a4a1b (T143C-C12063T-A15322G) (Fig. 2A). The Tuscan haplotype #66 belongs to a new branch within the new U7a4a1a clade.

The control region motif of U7a4 is easily searchable in the literature and public databases. Thus, in a large database of control region profiles, the HVS-I motif T16126C-A16309G-A16318T/A16318C has been found almost exclusively in the Near East and South Caucasus, including countries such as Iraq, Iran, Kuwait, Bahrain, Lebanon and Azerbaijan (S3 Table; Fig. 2B). This haplogroup has never been found in Europe, with the exception of the one instance observed in our Tuscans and two other in West Russia. The phylogeographic characteristics of this U7a4 point clearly to an origin in the Near East (Fig. 2C and S1 Fig.), not necessarily in Turkey, but most likely further to the East. It is also in the Near East where this haplogroup shows more variability (S1 Fig.). In addition, U7a4a1a is mainly found in the Near East.

The U7a4a1a clade is about 7.9 thousand years old (kya) (CI95%: 2.5–13.4) as estimated from the maximum likelihood procedure. The TMCRA estimated using the *ρ* statistic is also very similar (9.2 kya; CI95%: 3.1–15.6). Its origin therefore predates its presumable arrival to the Etruria. At the same time, it is noticeable that this clade has mostly evolved only locally in the Near East over such a long time period.

Sequence #60 belongs to a new clade of T2d2, named T2d2a (S2 Fig.). Within T2d2a there are two other mitogenomes, one coming from Italy (JQ798109) and the other one from Georgia (HM852899). In the root of T2d2 there are only two mitogenomes (not shown in S2 Fig.), one sampled in Iran (JQ798108) and the other one in Spain (JX415318).

There is another Tuscan mitogenome (#29) belonging to haplogroup J1b1a3a (S2 Fig; there is only another member of this clade in the literature that was sampled in Armenia, namely, JF286633). The most immediate ancestral node of J1b1a3a is represented by a mitogenome sampled in Italy (EF660916) and another one sampled in Iran (KC911610).

The Tuscan haplotype #24 belongs to T2n (S2 Fig.); this clade is represented by seven mitogenomes: two were found in Iran, indicating a possible origin of the Tuscan haplotypes in Near East, and three belong to the new T2n1 subclade and were sampled in Europe (one in Italy and two in Denmark).

There is another Tuscan haplotype (#95; S2 Fig.) that belongs to haplogroup J1d6; there are control-region defined haplotypes within this clade that have been suggested to be of Near East origin [8]. Curiously, when searching the EMPOP database for the motif A73G-T152C-A263G-C295T-C462T-T489C-C16069T-T16126C-C16193T (J1d), a high incidence of this haplogroup is identified in Near East: there are perfect matches in Dubai (*n* = 2), Uzbekistan (*n* = 2), Egypt (*n* = 1), and Iraq (*n* = 1). We observed a similar pattern when searching one-step mutation haplotypes (data not shown). There are three other mitogenomes belonging to J1d6, respectively sampled in Iran (KC911316), the Caucasus (Ossetia, JQ797894), and Republic of Tatarstan (GU122987).

The Tuscan haplotype #22 (S3 Fig.) falls into the H92 sub-clade, sharing the position T9497C with another Iranian mitogenome (Druze; EU600340) and suggesting a possible Near East origin for this clade. The control region motif of this haplotype is not very informative for database searches. However, when searching exact control region matches in EMPOP (rCRS motif of H92 plus T16368C) we observe a clear signal from Near East (three out of the seven matches were found in Iraq, Kuwait, and Arabia) and three from a linguistic Portuguese isolate (Miranda region), for which signatures from Near East have been reported [23]. Through literature searches of this sequence pattern we detected six additional haplotypes located in the South Caucasus region (*n* = 2, Armenia and Dagestan) [24], Lebanon (*n* = 1) [25], Iraq (*n* = 1) [26], Lyon (*n* = 1) [27] and the Latina province (*n* = 1) in Lazio (Central-West Italy) [28]. Taking all this evidence together, the Near East appears as the location that would best explain the origin and the geographic distribution of this clade.

Regarding haplotype #63 (S3 Fig.), this falls into a new haplogroup here named H97 (control region motif: A111G-T152C-T195C-A263G-T16209C-C16261T), and it was previously reported as exclusively shared between Tuscany and Near Eastern populations (control region motif T152C-T195C-A263G-T16209C-C16261T) [8]. No exact matches were found when performing searches of the control region haplotype T152C-T195C-A263G-T16209C-C16261T in EMPOP and the Sorenson databases; however, we observed several instances lacking the highly mutated transversion T195C: two haplotypes in North America, three in Palestine and one in Cyprus. In additional searches carried out on HVS-I segments in public resources (motif: T16209C-C16261T), we found one sample in Jordan [29], two in Turkey [24] and one in Austria [30]).

There is one Tuscan haplotype (#47; S4 Fig.) belonging to the sub-Saharan L1b1a haplogroup (more specifically belonging L1b1a5 haplogroup, here only defined by C14812T transition). This haplogroup has been reported to be of West-Central African origin [31] but some of its sub-clades could have originated and evolved within Europe in ancestral times [32]. There are two mitogenomes from Cyprus and one from Mauritania with the C14812T transition.

Finally, the rest of the mitogenomes observed in Tuscans are most likely of West European origin (S5 and S6 Figs.).

### Multidimensional scaling of Tuscan mitogenomes

**Fig 3:**
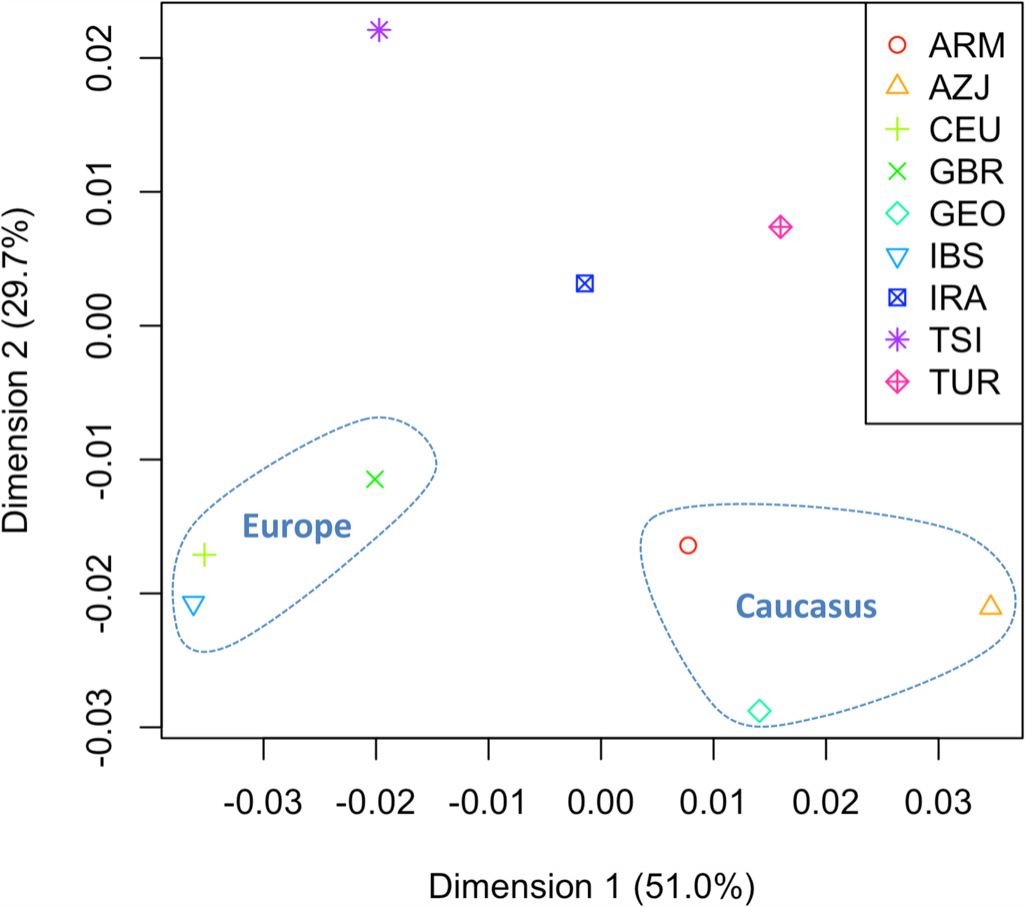
MDS of *F*_*ST*_ distances computed on mitogenomes. See S4 Table for more information on the population sets used and the *F*_*ST*_ values.

*F*_*ST*_ distances were computed on the few available mitogenome population datasets collected from the literature (S4 Table), and the distances were represented in a MDS plot (Fig. 3). The analysis reveals that the Dimension 1 (accounting for 51% of the variation) mainly differentiates the European from the Caucasus population sets; and Tuscans are more closely related to the European pole, while Iranians are closer to the Caucasus. However, in Dimension 2 (30% of the variation), the Tuscans appear more closely related to the Iranian population set than to Europe and the Caucasus, probably mirroring their proximity revealed by the phylogeographic analysis.

## Discussion

We analyzed the genetic characteristics of 110 Tuscan mitogenomes in the context of a large dataset of mitogenomes representing the worldwide phylogeny. There is strong evidence suggesting the existence of a Near East component in the Tuscans, thus adding further support to previous findings based on mtDNA control region data and autosomal data. If we consider #60 (T2d2), #29 (J1b1a3a), #24 (T2n1), #95 (J1d6), #92 and #105 (HV9c), #66 (U7a4a1a), #22 (H92) and #63 (H97) as haplotypes recently introduced to the Tuscan area from the Near East, the introgression of Near East haplotypes would account for 8.2% of the total mtDNA Tuscan pool. This signal is significantly lower than the one observed at a genome-wide scale (21%). Moreover, the autosomal data indicate that carriers of Near East mitogenomes do not correspond to migrants arriving recently to Tuscany from the Near East (S1 Text).

Within the Near East, the main genetic signature comes from Iran, although this view could be distorted by an overrepresentation of this region in the database of mitogenomes from the Near East. The genetic link between Tuscans and other Near East populations (e.j. Syria, Armenia) is in good agreement with the analyses of admixture carried out by Hellenthal et al. [33] based on autosomal markers. The second most important signal in our study would come from South Caucasus. Note that an origin in South Caucasus of the Near East component of Tuscans would also fit well with the findings observed in the genome-wide SNP analysis carried out by Pardo-Seco et al. [11].

Haplotypes of presumable Near East origin represent different clades of the mtDNA phylogeny. This feature suggests a demographic scenario involving a moderate number of Near East immigrants into Tuscany (thus explaining the lack of evidence for founder effects) and a relatively recent arrival to the region. Such a scenario would be also compatible with local bottlenecks in isolated populations in Tuscany, as is the case of haplogroup U7b1 in the Isle of Elba (Tyrrhenian Sea, Tuscany) [3]. Given that all the Tuscan haplotypes of Near East origin represent isolated members of different clades, it is not possible to date the arrival of this Near East mitogenomes.

The present study adds further support to previously reported findings suggesting the presence of a significant Near East component in Tuscan mitogenomes, and points to Iran as the region in the Near East providing the main genetic signal to present day Tuscans.

## Supporting Information

S1 Table Complete Tuscan mitogenomes used in the present study. In addition, we have also compared the differences between the annotated variants reported by Zheng et al. [14] using the same dataset.
(XLSX)

S2 Table Raw data as directly obtained from the annotated software. Indels were re-annotated manually in order to accommodate the nomenclature to standards in mtDNA studies.
(XLSX)

S3 Table List of mitogenomes of haplogroups HV9 and U7a4 used to build the trees of Figs. 1 and 2 as obtained from public sources. Control region data, frequencies and diversity values of U7a4 mtDNAs are also provided.
(XLSX)

S4 Table Information on the mitogenomes used for computing *Fst* distances and the matrix of pairwise *Fst* values. (XLSX)

S1 Fig Map showing the geographic distribution of haplogroup U7a4 interpolated nucleotide diversities. (TIF)

S2 Fig Maximum parsimony tree of haplogroups J and T of Tuscan mitogenomes and their closest matches found in public sources. For details see caption to Fig. 1.
(TIF)

S3 Fig Maximum parsimony tree of haplogroups H(×H1) of Tuscan mitogenomes and their closest matches found in public sources. For details see caption to Fig. 1.
(TIF)

S4 Fig Maximum parsimony tree of haplogroups X, W, D and sub-Saharan L of Tuscan mitogenomes and their closest matches found in public sources. For details see caption to Fig. 1.
(TIF)

S5 Fig Maximum parsimony tree of haplogroups H1 of Tuscan mitogenomes and their closest matches found in public sources. For details see caption to Fig. 1.
(TIF)

S6 Fig Maximum parsimony tree of haplogroups U of Tuscan mitogenomes and their closest matches found in public sources. For details see caption to Fig. 1.
(TIF)

S1 Text Estimates of autosomal ancestry of Tuscans based on autosomal SNPs (>540.000; see Pardo-Seco et al. [11] for details) and their mtDNA haplogroup adscription. (DOC)
