# Supplementary figures and images for: Mitogenomes from The 1000 Genome Project Reveal New Near Eastern Features in Present-Day Tuscans
Source: PLoS One. 2015 Mar 18;10(3):e0119242. doi: 10.1371/journal.pone.0119242 (PMC4365045; doi:10.1371/journal.pone.0119242)

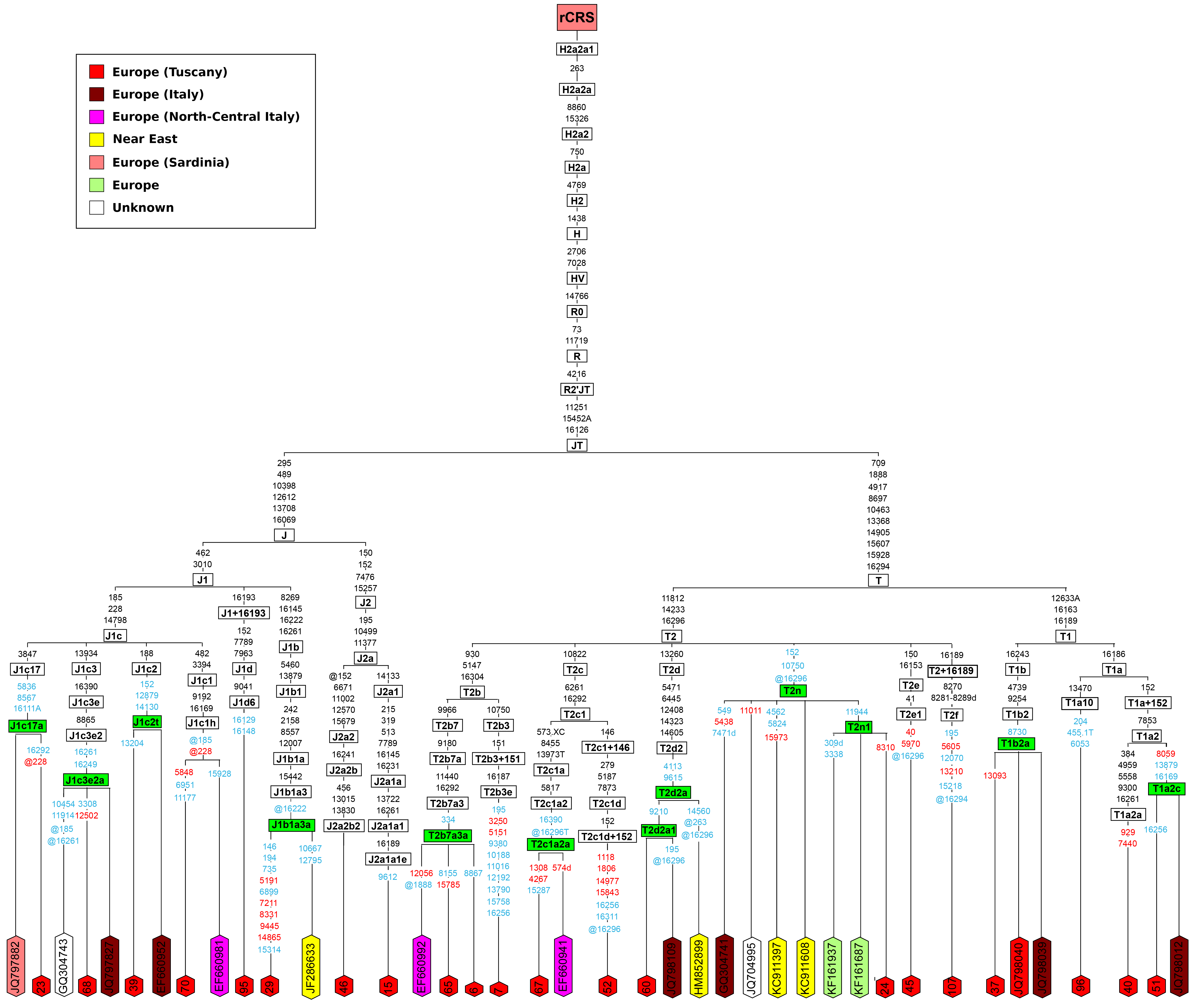

Supplement: S2 Fig — For details see caption to Fig. 1. (TIF) [file pone.0119242.s006.tif]

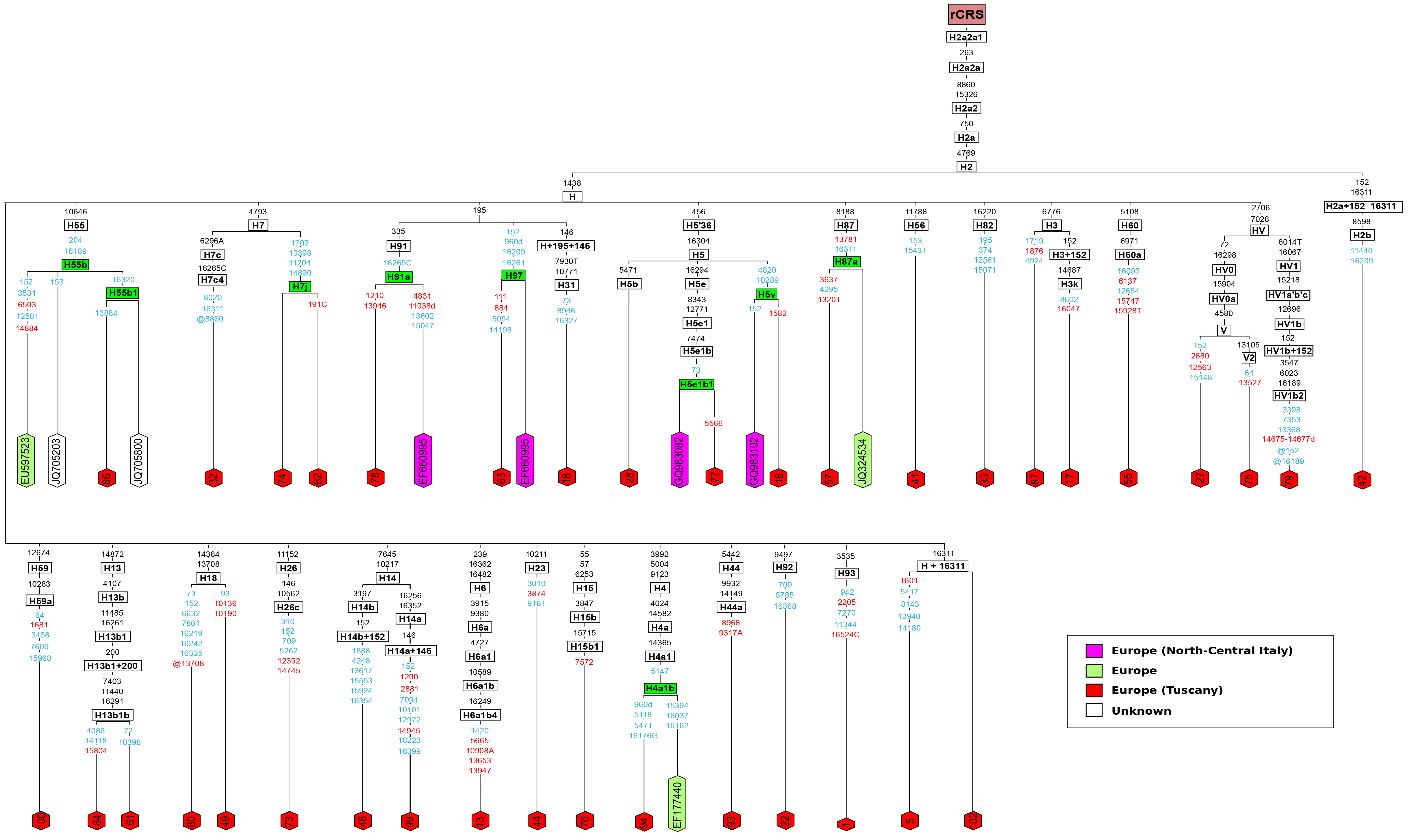

Supplement: S3 Fig — For details see caption to Fig. 1. (TIF) [file pone.0119242.s007.tif]

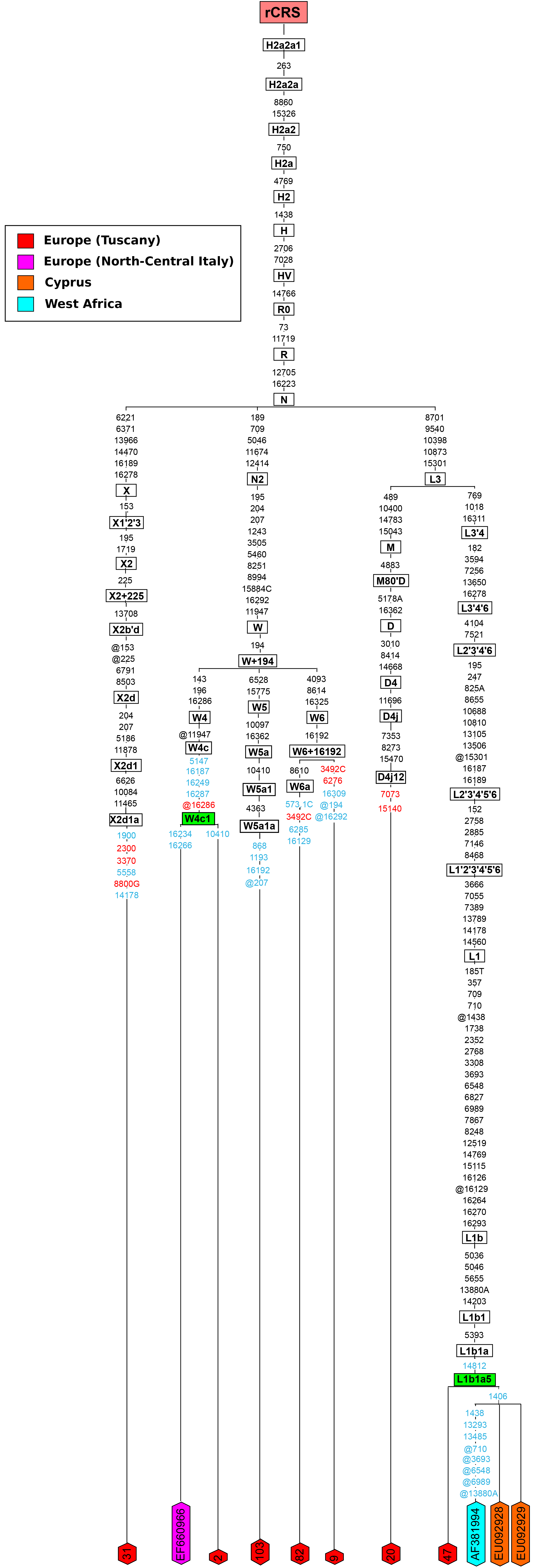

Supplement: S4 Fig — For details see caption to Fig. 1. (TIF) [file pone.0119242.s008.tif]

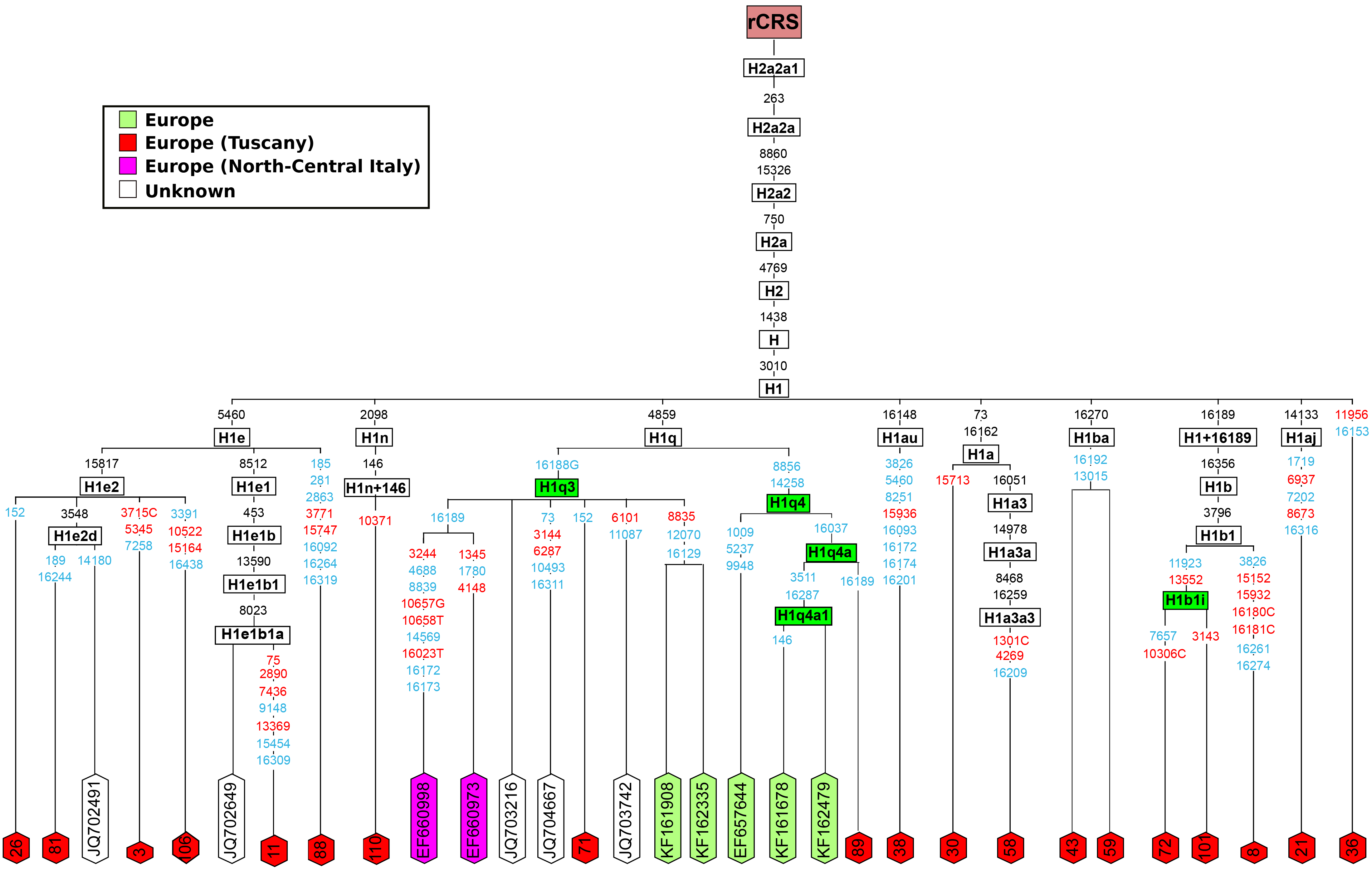

Supplement: S5 Fig — For details see caption to Fig. 1. (TIF) [file pone.0119242.s009.tif]

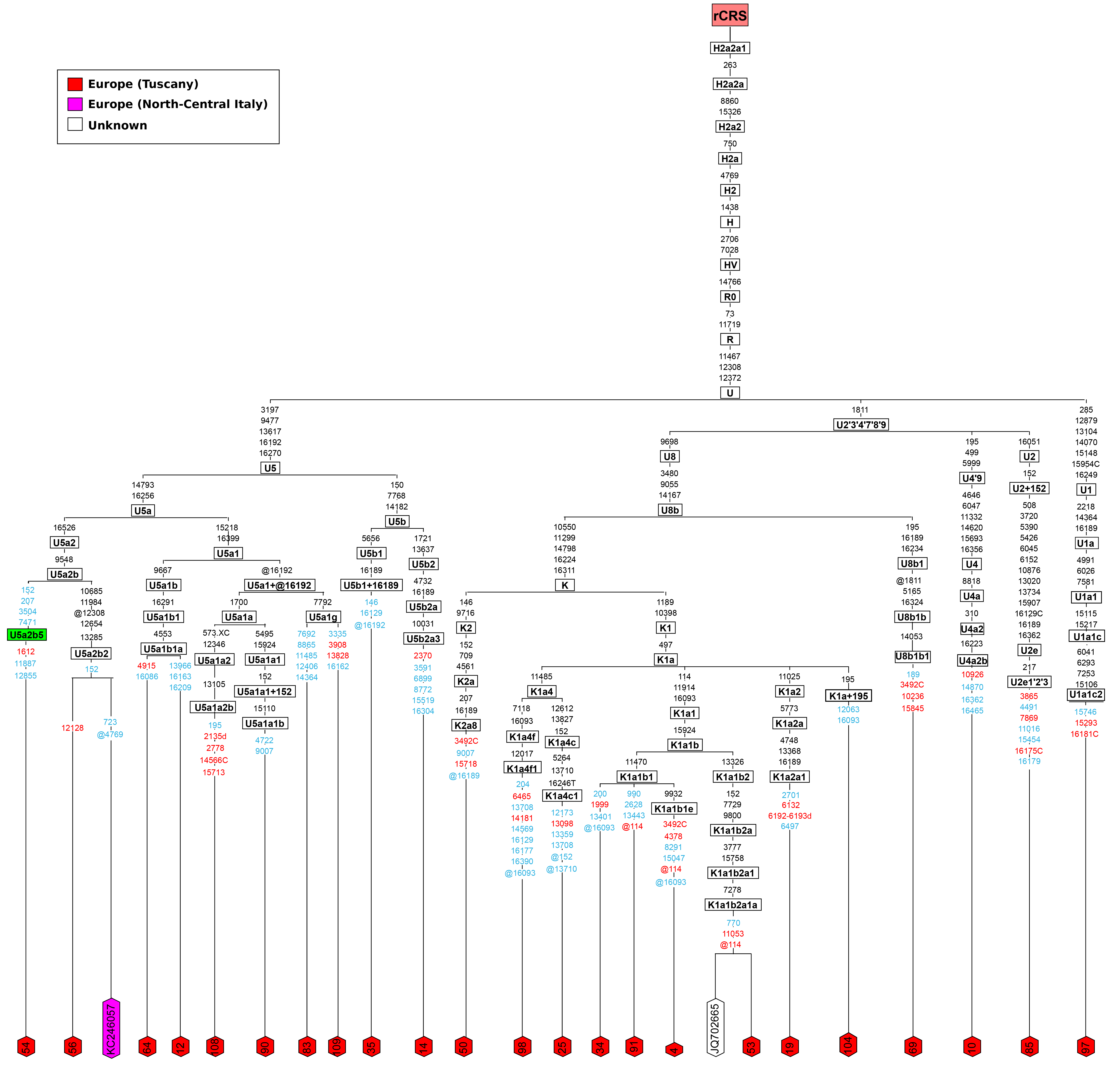

Supplement: S6 Fig — For details see caption to Fig. 1. (TIF) [file pone.0119242.s010.tif]
