# Supplementary material for: Mitogenomes from The 1000 Genome Project Reveal New Near Eastern Features in Present-Day Tuscans
Source: PLoS One. 2015 Mar 18;10(3):e0119242. doi: 10.1371/journal.pone.0119242 (PMC4365045; doi:10.1371/journal.pone.0119242)
Supplement: S1 Text — (DOC) [file pone.0119242.s011.doc]

**Text S1.**

**Correlation of mtDNA haplotypes and estimates of ancestry based on autosomal data**

Analyses of autosomal SNP data of Tuscans were carried out in Pardo-Seco et al. . Here we just correlate Tuscan mtDNA haplotypes and their haplogroup adscription with the membership ancestry estimates of these Tuscans in Near East and Europe. As explained in Pardo-Seco et al. , the CEU SNP dataset from The 1000 Genome Project (representing a population of European ancestry) and the Near East SNP data published by Behar et al. were used as surrogate ancestral populations for admixture analysis.

The methodology used is explained in detail in Pardo-Seco et al. . In brief, identity-by-state (IBS) values were computed from autosomal SNP data using PLINK . ADMIXTURE software was used to estimate percentages of admixture in Tuscans considering Europe and Near East as the main source ancestral populations; this software uses a maximum likelihood estimation of individual ancestries from multilocus SNP data.

As shown in the Figure below, all Tuscans have a main European component (represented by CEU) that is on average 79% (95%CI: 78.7-79.4). The Near East component in Tuscans is 21% on average (95%CI: 20.6-21.3). As expected, there is no particular correlation between Tuscan mitogenomes of likely recent Near East origin and the proportion of Near East ancestry measured on the autosomes.


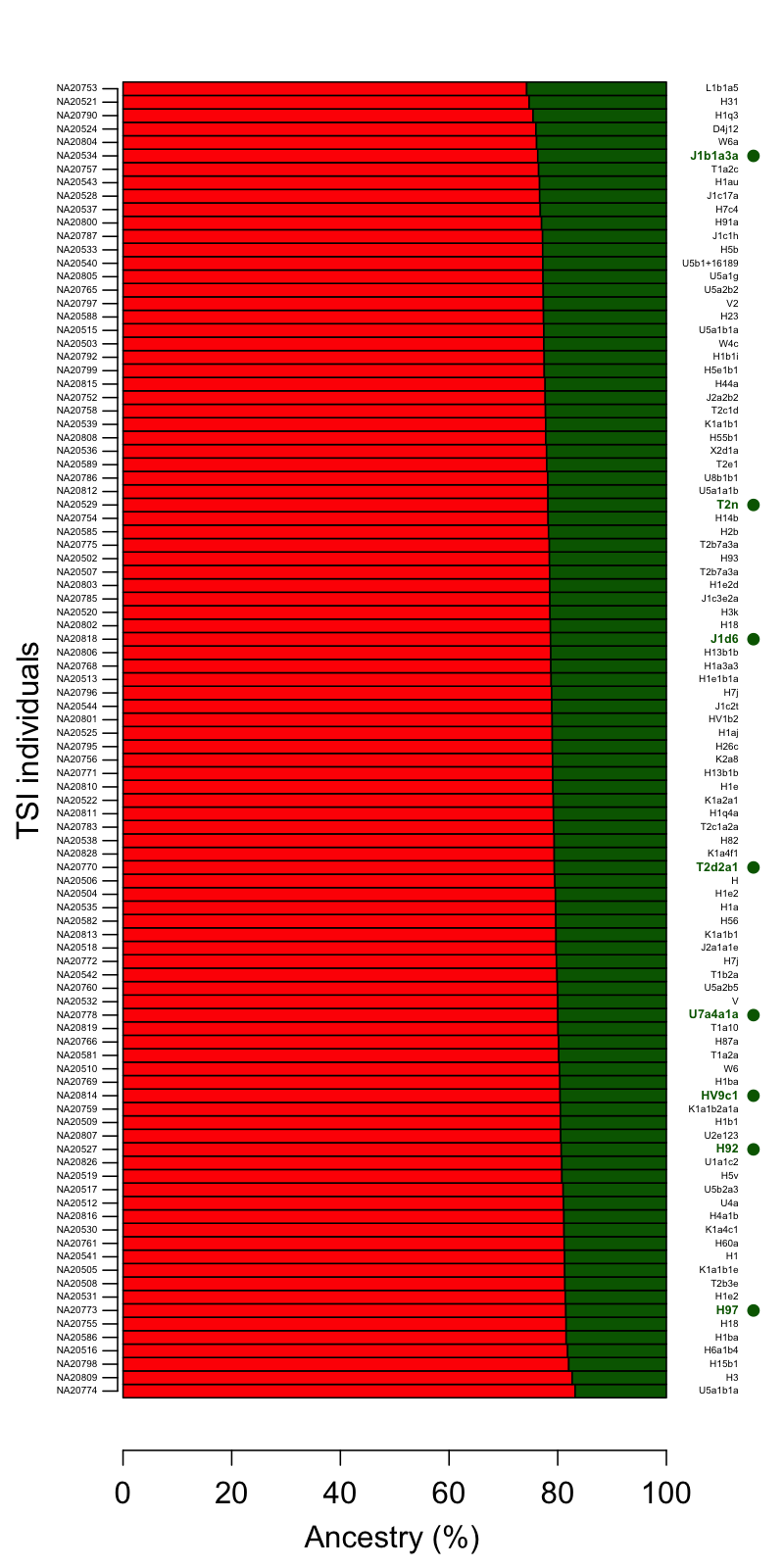


**Figure.** Ancestry estimates in Tuscan individuals as inferred from autosomal data. Admixture analysis for *K* = 2; see Pardo-Seco et al. for details. Red color represents the membership ancestry that predominates in Europeans, while green represents the ancestry that is predominant in Middle East. On the right are the mtDNA haplogroup ascriptions of Tuscan haplotypes; green dots point to those mtDNA that are more likely to be of recent Middle East origin as described in the main text.

**References**

1. Pardo-Seco J, Gómez-Carballa A, Amigo J, Martinón-Torres F, Salas A (2014) A genome-wide study of modern-day Tuscans: revisiting Herodotus’s theory on the origin of the Etruscans. PLoS One 9: e105920.

2. Behar DM, Hammer MF, Garrigan D, Villems R, Bonne-Tamir B, et al. (2004) MtDNA evidence for a genetic bottleneck in the early history of the Ashkenazi Jewish population. Eur J Hum Genet 12: 355-364.

3. Purcell S, Neale B, Todd-Brown K, Thomas L, Ferreira MA, et al. (2007) PLINK: a tool set for whole-genome association and population-based linkage analyses. Am J Hum Genet 81: 559-575.

4. Alexander DH, Novembre J, Lange K (2009) Fast model-based estimation of ancestry in unrelated individuals. Genome Res 19: 1655-1664.
